# Aberrant Development of Functional Connectivity among Resting State-Related Functional Networks in Medication-Naïve ADHD Children

**DOI:** 10.1371/journal.pone.0083516

**Published:** 2013-12-26

**Authors:** Jeewook Choi, Bumseok Jeong, Sang Won Lee, Hyo-Jin Go

**Affiliations:** 1 Department of Psychiatry, Daejeon St. Mary’s Hospital, The Catholic University of Korea, College of Medicine, Daejeon, Republic of Korea; 2 Laboratory of Clinical Neuroscience and Development, Graduate School of Medical Science and Engineering, Korea Advanced Institute of Science and Technology, Daejeon, Republic of Korea; University of Electronic Science and Technology of China, China

## Abstract

**Objective:**

The aim of this study was to investigate the compromised developmental trajectory of the functional connectivity among resting-state-related functional networks (RSFNs) in medication-naïve children with attention-deficit/hyperactivity disorder (ADHD).

**Subjects and Methods:**

Using both independent component analysis and dual regression, subject-specific time courses of 12 RSFNs were extracted from both 20 medication-naïve children with ADHD, and 20 age and gender-matched control children showing typical development (TDC). Both partial correlation coefficients among the 12 RSFNs and a resting-state resource allocation index (rsRAI) of the salience network (SN) were entered into multiple linear regression analysis to investigate the compromised, age-related change in medication-naïve ADHD children. Finally, correlation analyses were performed between the compromised RSFN connections showing significant group-by-age interaction and rsRAI of SN or clinical variables.

**Results:**

Medication-naïve ADHD subjects failed to show age-related increment of functional connectivity in both rsRAI of SN and two RSFN connections, SN-Sensory/motor and posterior default mode/precuneus network (pDMN/prec) – anterior DMN. Lower SN-Sensory/motor connectivity was related with higher scores on the ADHD Rating Scale, and with poor scores on the continuous performance test. The pDMN/prec-aDMN connectivity was positively related with rsRAI of SN.

**Conclusions:**

Our results suggest that medication-naïve ADHD subjects may have delayed maturation of the two functional connections, SN-Sensory/Motor and aDMN-pDMN/prec. Interventions that enhance the functional connectivity of these two connections may merit attention as potential therapeutic or preventive options in both ADHD and TDC.

## Introduction

Abnormalities beyond the fronto-striatal circuit in attention-deficit/hyperactivity disorder (ADHD) subjects have been consistently reported in recent neuroimaging studies. Brain abnormalities have been found not only in the frontal-striatal circuitry [1], [2] but also in other brain regions including the occipital, parietal [3], temporal, and default mode network (DMN) in ADHD subjects [4]–[6]. The aberrant connection were found among functional brain networks, for example, within-[7]–[9] and between-DMN [10], the dorsal anterior cingulate cortex (dACC)-DMN [11], [12], and intra- and extra-regional connectivity of the dorsal attention, cerebellum and reward-motivation regions [8]. These results are in line with the extended conceptualization of ADHD beyond simply aberrant fronto-striatal functional connections. On the basis of previous neuroimaging studies, Castellanos and Proal, recently, proposed the involvement of large-scale brain systems beyond the prefrontal-striatal model in ADHD [13]. They introduced seven macro-scale, functional brain networks including the fronto-parietal, dorsal attentional motor, visual and default mode networks; then described abnormalities in each network in cases with ADHD [13]. They also proposed investigation of the interaction among candidate functional networks which can form distinguishable neurobiological patterns [13].

In developing children, however, age is an important factor to consider when exploring the brain as a network system. A recent study reported that cortical thinning across age was found in bilateral hemisphere of both ADHD and healthy subjects, when comparing children to young adults [5]. Furthermore, ADHD in childhood can continue into adolescence and adulthood [14]. Some neuroimaging studies reported developmental abnormality in those with ADHD. For example, the strength of causal regulatory influences from the anterior insula (AI) to the posterior parietal cortex (PPC) node of the central executive network (CEN) was significantly weaker and contributed to lower levels of behavioral performance in children with ADHD, compared to adults with ADHD [15]. In the study with a multivariate machine-learning approach, compared to adult controls, adults with ADHD showed decreased dACC-DMN, but were not different from young controls [16]. According to the DMN Interference Hypothesis postulated by Sonuga-Barke and Castellanos [17], attentional lapses, temporary shifts of conscious attention away from the primary task to unrelated internal information processing, is related to the aberrant interaction between the task-negative DMN, and the task-positive attentional network, in ADHD subjects [18]. Previous studies have suggested that the aberrant interaction among AI, CEN, DMN may represent attentional lapses and have developmental properties.

To explore the brain as a network system in developing children, there are some considerations for constructing functional networks, such as the selection and the number of regions of interest (ROI), and the method for measuring functional connectivity. One method is the selection of ROIs derived from either anatomical parcellation in each subject, or from use of anatomical atlases. While this method can show the characteristics of large-scale networks of the brain system, the lack of functional meaning of anatomy-based ROIs is the most critical disadvantage. In the present study, the method for choosing ROIs was the extraction of macro-scale functional networks with a data reduction method such as independent component analysis (ICA), a multivariate method. The method using ICA can reduce brain imaging data, without any significant data loss, into several or tens of macro-scale functional networks which can better reflect functionality than do anatomical-atlas-based approaches. If ICA is performed with low number of dimensions, a brain network can be represented, and be understood, with the interactions among a small number of the functional ROIs. Here, using low dimension ICA-dual regression analysis for resting-state fMRI data from medication-naïve ADHD subjects, we delineated subject-specific functional networks and their time courses. Then, partial correlation was used to measure functional connectivity, which controls the effect of other connections on a specific connection, among the resting-state functional networks (RSFNs).

For the theory-driven test, our approach considering interactions among all networks in brain can provide less biased results than the previous method without considering them [11], [12]. Here, to investigate the developmental abnormality of CEN-SN-DMN connectivity, we proposed a resource allocation index in resting state (rsRAI) which was obtained by subtraction from SN-CEN to SN-DMN connectivities. Low rsRAI means that subject’s attention can easily shift to internal situation and may represent attentional lapses.

In sum, previous studies have suggested that brain abnormalities in those with ADHD are not only in the fronto-striatal circuit, but also involve interaction among whole brain functional networks, the salience network (SN) in particular, including dACC, AI and PPC, CEN, and DMN. The altered interaction among SN, CEN, and DMN may be related with attentional lapses in ADHD. Also, the developmental properties may influence the aberrant interactions among functional networks. However, to the best of our knowledge, no study has dealt with considerations of the aspects of development of macro-scale whole brain systems, especially using such as functional ROI selection, connectivity measures, rsRAI and developmental properties; simultaneously, in medication-naïve ADHD children.

We test following hypotheses. First, medication-naïve ADHD have the aberrant development of specific functional connections in whole brain network consisting of several or tens of RSFNs. The aberrant connectivities may relate with behavioral and cognitive measures. Secondly, based on the theory from previous studies, rsRAI of SN also shows the aberrant development in medication-naïve ADHD children. Finally, there are the association of the aberrant development of functional connections and rsRAI showing significant age by group interaction, implicating underlying mechanism in ADHD brain network.

## Methods

### Subjects

We recruited psychotropic medication-naïve children with ADHD, aged 6 to 16 years, by advertisements targeting children who visit the child and adolescent psychiatric clinic in St. Mary’s Hospital (Daejeon, South Korea). Typically-developing control children (TDC) were also recruited by advertisements targeting children who were between 6–16 years of age, and who had no history of psychiatric disorder. The Diagnostic and Statistical Manual of Mental Disorders (Fourth Edition - DSM-IV) criteria on structured diagnostic interview (Kiddie-Schedule for Affective Disorders and Schizophrenia-Present and Life time Version-Korean Version) were used for diagnosis. All subjects were right-handed and had full-scale Intelligence Quotients above 70. Subjects were also required to be free from any neurological disease or insult, developmental disorders including pervasive developmental disorders or mental retardation, and medical disorders that could affect brain development. Subjects were excluded who had any history of substance abuse, any recent substance use, psychotropic medication, head trauma, significant fetal exposure to alcohol or drugs, and perinatal or neonatal complications. Subjects were excluded with claustrophobia, irremovable metal materials, or poor cooperation with the MRI scanning procedure. The Catholic University of Korea – Daejeon, St. Mary’s Hospital Institutional Review Board approved all procedures. The purpose of the study was explained to subjects and parents, who gave their written informed consent. All parents provided demographic information (Table 1). The number of subject was evenly distributed across age ranges (supplemental table S3).

**Table 1:** Subject Characteristics.

	ADHD (n = 20)	TDC (n = 20)
Age, y	10.2±2.7	10.6±2.5
Height, cm	141.8±14.6	144.4±12.5
Weight, kg	40.4±15.0	42.0±10.9
Full scale IQ**	95.0±11.5	114.6±12.6
Performance**	92.8±12.5	111.2±14.3
Verbal*	97.8±11.4	113.1±14.7
Father’s education, y	14.7±2.2	14.7±2.2
Mother’s education, y	13.7±2.0	14.5±2.0
Korean ADHD Rating Scale**	38.1±8.2	6.2±4.1

P<.05,

P<.001.

### Clinical and Cognitive Measures

ADHD and other psychiatric comorbidities were determined based on the Korean Kiddie-Schedule for Affective Disorders and Schizophrenia-Present and Lifetime Version (K-SADS-PL), a semi-structured diagnostic instrument. The reliability and validity of the K-SADS-PL has been previously determined [19]. The full-range of The Korean Educational Development Institute-Wechsler Intelligence Scales for Children (KEDI-WISC) III, was administered to each subject to assess intellectual functioning.

For assessment of ADHD symptoms, inattentiveness and hyperactivity were evaluated by both parents and psychiatrists based on the Korean version of the ADHD rating scale (K-ARS). The severity of ADHD symptoms were assessed with the ADHD Rating Scale (ARS) which was introduced by Dupaul and his colleagues [20]. The ARS contains 18 items; nine of these assess inattention, and nine are related to hyperactivity and impulsivity. The K-ARS form is considered to have high validity and reliability [21]. Korean Conner’s Parent Rating Scale (K-CPRS) was used to assess ADHD symptoms.

Assessment of neurocognitive functions has also been done. A computerized, continuous performance test (CPT) [22], [23] was used to measure cognitive functions. The four major variables recorded were (1) omission errors, (2) commission errors, (3) response times, and (4) the standard deviations of the response times for correct responses to the target (response time variability). Here, we used only visual, not auditory, CPT for 15 minutes to each child for time efficiency. For working memory tests, we used Digit Span in the KEDI-WISC III [24] to assess auditory working memory function, and the Finger Window Test for the visual working memory function. The COWAT is one of the verbal fluency tests, which reflects another executive function. We tested both semantic fluency and phonemic fluency of each subject. All of the data are presented as T-scores adjusted for age and gender. Higher T-scores indicate better function in all tests except the CPT, in which lower T-scores indicate better attention and response-inhibition ability.

### fMRI Data Acquisition

Using a 1.5 T Philips scanner at Daejeon St. Mary’s Hospital, fMRI imaging was acquired with a BOLD-sensitive echo-planar gradient-echo (EPI) sequence with the following imaging parameters: repetition time (TR): 2000 ms; echo time (TE): 35 ms; flip angle (FA): 70°; spatial resolution: 3.4375×3.4375×5 mm^3^; imaging matrix: 64×64; field-of-view (FOV): 240×240 mm^2^, number of slices: 27; 210 volumes. Subjects were instructed to keep their eyes closed and not to think of anything particular during fMR scans. A T1-weighted anatomical image was also acquired using a magnetization-prepared, gradient-echo sequence (TR = 25 ms; TE = 4.6 ms; 170 slices; FOV = 240×240 mm^2^; voxel size = 1×1×1 mm). The head was stabilized with cushions and taped to help minimize movement.

### Data Preprocessing

The fMRI data were processed and analyzed with the MELODIC (Multivariate Exploratory Linear Optimized Decomposition into Independent Components) module of the FSL software (http://www.fmrib.ox.ac.uk/fsl/feat5/index). The first five scans were discarded to account for T1 saturation effects. The remaining 205 images were spatially realigned using rigid-body transformation and were subjected to slice-timing correction. Next, a brain mask from the first fMR data volume was created to eliminate signals outside of the brain, for each subject. Then, a temporal band-pass filter (0.01 Hz<f <0.1) was applied to effectively remove signal drift and to get a resting-state-related signal. Spatial smoothing using a Gaussian kernel of full-width at half-maximum 6 mm was performed to reduce noise without reducing valid activation. The serial correlations were removed to make the statistics valid and maximally efficient. Finally, corresponding fMRI volumes were registered with corresponding 3D high-resolution structural images that had been co-registered to the Montreal Neurological Institute (MNI) T1 template using FLIRT (FMRIB’s Linear Image Registration Tool) of FSL. The fMR volumes were transferred into the MNI space with 4-mm isotropic resolution.

### Estimation of Subject-specific Time Course and Associated Spatial Map for Each Independent Component using ICA and Dual Regression

**Figure 1:**
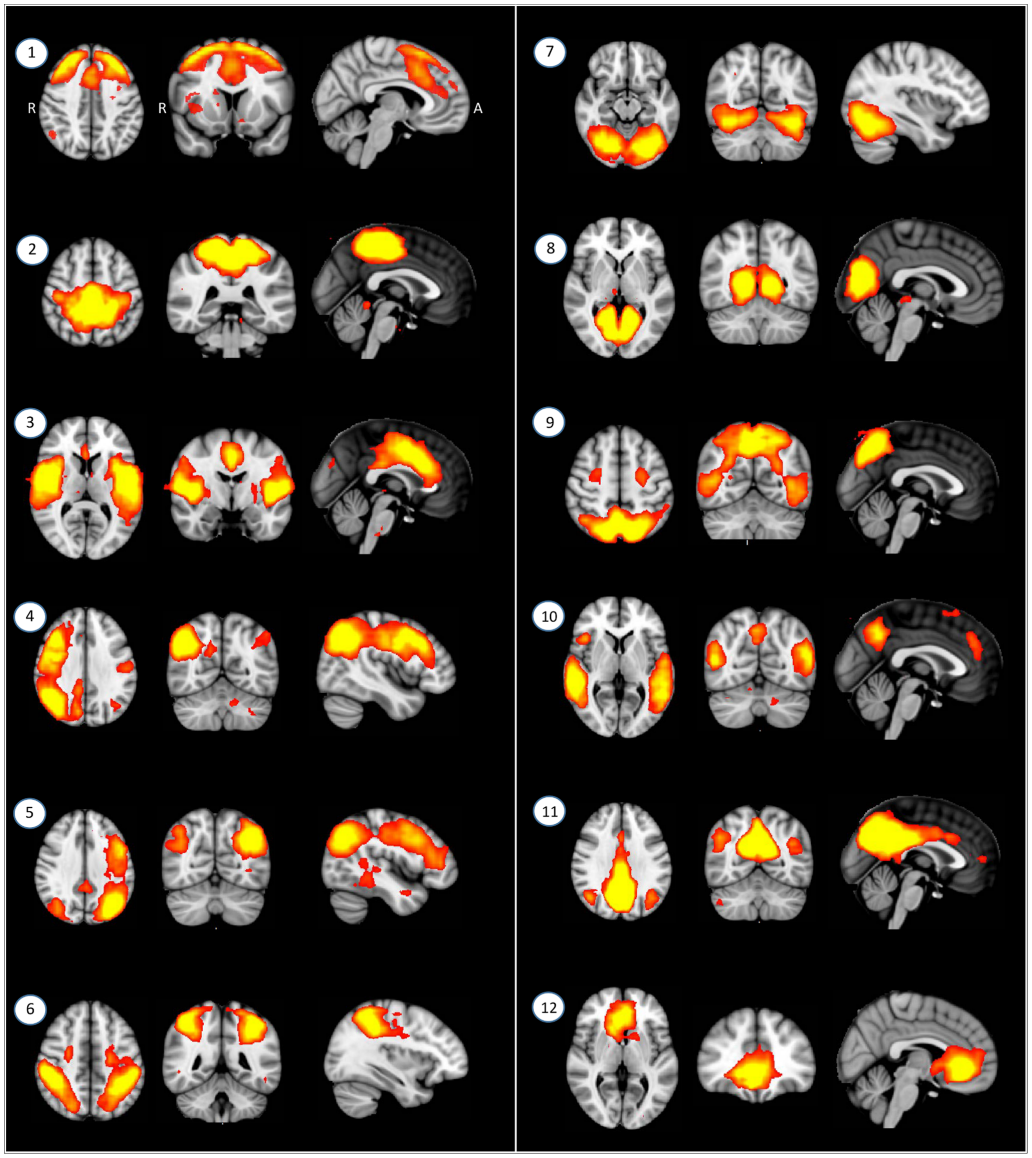
Twelve spatially independent resting-state-related functional networks (RSFNs). From left-upper to right-lower part: RSFN1: Frontal; RSFN2: Sensory/motor; RSFN3: Salience (SN, Ventral attentional); RSFN4: Right central executive (rCEN); RSFN5: Left central executive network (lCEN); RSFN6: Dorsal attentional (dAtt); RSFN7: V1; RSFN8: V1/V2; RSFN9: Extrastriate; RSFN10: a temporooccipital part of posterior DMN (pDMN/TO); RSFN11: a precuneus part of Posterior default mode (pDMN/prec); RSFN12: Anterior default mode (aDMN). Radiologic orientation (left is right). MNI coordinates of RSFNs were presented in Table S1.

At first, spatially independent resting-state-related functional networks (RSFNs) in the population of all subjects were identified using MELODIC. This involves: (i) using a standard (subject×space×time) ICA decomposition of the temporally concatenated [25] dataset of all 40 participants as a group, resulting in 28 spatially independent components; and (ii) using a Gaussian/gamma mixture-model of the distribution of voxel intensities within spatial maps and an alternative hypothesis testing approach at a threshold level of 0.5. This resulted in threshold-group-ICA spatial maps exceeding the probability of its being background-noise [26]. Secondly, after removal of 16 artifacts (e.g., residual motion, mal-alignment, non-gray matter signals: supplemental Figure S1) from 28 independent components, there remained 12 RSFNs (Figure 1) for more detailed connectivity analyses. Finally, the subject-specific temporal dynamics of each RSFN were identified using dual, spatial and temporal, regressions. In spatial regression, matrices representing temporal dynamics for each component and subject were produced using the full set of group-ICA spatial maps in a linear model fit against the separate fMRI data set [27]. In the temporal regression, subject-specific spatial maps were estimated using the time course of each component and subject. Additionally, ICA was performed for each group as running separate ICA may avoid the specific RSFN pattern from each group [28]–[30]. Spatial correlation analyses between corresponding RSFNs from each group showed that spatial patterns of RSFNs were consistent (figure S2). Thus, RSFNs resulted from combined group ICA were used in next step.

### Estimation of Correlation Coefficients among 12 Subject-specific RSFNs

Functional connectivity analyses were performed using time courses of the 12 RSFNs of each subject, which were acquired with dual regression. Results from a functional connectivity study using simulated fMRI data showed that partial correlation (PC) analysis was more reliable than cross correlation (CC) analysis [31]. We also reported that the functional connectivity among RSFNs was more consistent using PC than CC [32]. A possible indirect effect of functional connectivity among other RSFNs, on the functional connectivity of a certain RSFN pair, can be controlled-for using PC analysis. The 16 artifactual, independent components were also regressed out in the correlation analysis. Thus, PC was used as a measure of functional connectivity in the present study. This PC analysis produced PC matrices of 12 by 12 for each subject. The PC referred to the covariance between time courses of members in each RSFN pair. Fisher’s r-to-z transformation was performed on the partial correlation coefficients. For group mean correlation matrices, z-to-r transformation was performed on the group-mean z-transformed correlation coefficients (Table S2).

### Age-by-group Interaction Using Multiple Linear Regression Analysis for All Possible Functional Connections

As brain development is very active from age 7 to 16, the functional connectivity should also be actively changing during childhood and adolescence [33], [34]. Both IQ and age should be also considered. Thus, we hypothesized that functional connectivity (FC) at each RSFN pair may be estimated with group, age, IQ, gender and group-by-age interaction. The z-transformed correlation coefficient of each RSFN pair was used in the multiple linear regression analysis with the ‘lm’ function of R package (http://stat.ethz.ch/R-manual/R-patched/library/stats/html/lm.html). The linear regression model proposed was:
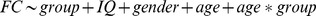



For the correction for multiple comparisons (66 pairs), false discovery rate (FDR) was performed for each main effect or interaction separately, at a statistical threshold level of q <0.05. To confirm whether our proposed model is apt or not, the model was selected using the bidirectional elimination approach for each surviving RSFN pair. Then, the proposed model was compared with the selected model by bidirectional elimination approach and with the non-interaction model (FC ∼ group+IQ+gender+age). If there was no significant difference, the simpler model was chosen as the best model. All multiple linear regression analyses were performed using ‘MASS 7.3’ of ‘R package’ (http://cran.r-project.org/web/packages/MASS/index.html). If the model selected by bidirectional elimination approach was the best model, FDR was recalculated with the p-value of the selected model. In each model, both normal distribution and skewedness were tested with the Shapiro-Wilk normality test. The confidence interval of each parameter was calculated using bootstrapping with ‘boot’ of ‘R package’ because the sample size (n = 40) was small in the present study. To compare the slope of the group-by-age interaction after having adjusted other factors, multiple linear regression analysis was performed on each group and the slope for each group was displayed using added-variable (partial regression) plot An alternative connectivity analysis is the network-based statistics NBS (Zalesky et al., 2010), which is known as a method for coarse sampling resolution (Zalesky et al., 2012) as was present in this study. Although the NBS is a nonparametric statistical test and defines clusters using the graph theoretical concept of connected component, it has the disadvantage that it identifies two separate pairwise connections (B–A and B–C) as one cluster (A–B–C). In a recent ADHD study using NBS, for example, altered intrinsic connectivity in many regions such as orbitofrontal-temporal-occipital and fronto-amygdala-occipital network clusters, was reported (Cocchi et al., 2012). Thus, if the developmental property is confined to a certain functional connection, our approach might be more suitable than NBS to detect it. Permutation method is another option for functional neuroimaging data which are used for inference when the null distribution is not known [35]. Although the permutation method is also a nonparametric statistical test, it has the disadvantage that it is not easily applied for complicated model with two or more interaction terms. Finding the best fitted model is also not easy with the permutation method. In contrast to permutation method, our approach have advantages for finding the best fitted model if we cannot expect that all functional connections are explained with same model.

### Age-by-group Interaction Using Multiple Linear Regression Analysis for Resource Allocation Index in Resting State (rsRAI)

The salience network (SN) including the anterior insula (AI) and anterior cingulate cortex (ACC), plays a critical role in switching between the DMN and the CEN (Menon et al., 2010; Sridharan et al., 2008). SN may allocate brain resources to either CEN or DMN depending on internal and external situations. Terminology of ‘resource allocation’ having been used when overactivation of brain regions was also linked to the modulation of intrinsic activity of DMN [36], [37]and to its functional connectivity with executive network [38], [39]. In this study, we hypothesize this resource allocation by the SN may have not only developmental property but also difference in the slope across age between ADHD and TDC groups. The resource allocation index in resting state (rsRAI) was calculated using:
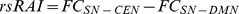



Here, FC_SN-CEN_ and FC_SN-DMN_ indicate differences in partial correlation coefficients between SN and CEN; and SN and DMN, respectively. Four rsRAIs were calculated (i.e., left CEN-SN-aDMN, right CEN-SN-aDMN, left CEN-SN-pDMN/prec, and right CEN-SN-pDMN/prec). High rsRAI may represent the status for getting ready to cope with external stimuli in resting-state and may be related to a low possibility of attentional lapses. We hypothesized that resource allocation by SN may have developmental properties in TDC which may not be shown in medication-naïve ADHD children. To test our hypothesis, age-by-group interaction was investigated for each rsRAI using multiple linear regression analysis.

### Relationship between Functional Connectivity and Clinical Variables

The functional connection among RSFNs may be related to various phenotypes of ADHD regarding behavior and cognitive ability. Here, using ‘Spearman’s rank order partial correlation analyses’, we explored the relationship between functional connectivity of RSFN pairs, or rsRAI, showing a significant group-by-age interaction and clinical variables after controlling out the effect of age, IQ and gender; at the level of P<0.05. The relationship between IQ and functional connections of RSFN pairs was also explored with the effect of age and gender controlled out at the level of P<0.05.

## Results

### Demographical and Clinical Findings

Twenty medication-naïve children with ADHD (mean age 10.2±2.7) and 20 age and gender (M:F = 16∶4) matched TDC (mean age 10.6±2.5) were included in this analysis. The two groups did not differ in age, height, weight, paternal or maternal education levels, but the ADHD-group showed lower scores on the Full Scale IQ Score than the TDC group, and significantly higher scores than TDC groups of the ADHD Symptom Rating Score (P<0.001 - Table 1). Among the 20 subjects in the ADHD group, fifteen subjects were classified as having the combined-type of ADHD and the others were of the inattentive-type. Five subjects (25%) of the ADHD group showed other psychiatric comorbidities (three with oppositional defiant disorder, one with depressive disorder, one with social phobia) on structured diagnostic interview (K-SADS-PL). The ADHD group, compared with TDC, showed higher ADHD symptom scores such as K-ARS and CBCL scores, and also performed more poorly on the CPT (omission error, commission error, standard deviation of response time); digit span (backward and total score); and finger window (forward, backward, total score) tests (Table 2).

**Table 2:** Behavioral and cognitive symptoms.

	TDC	ADHD	t	df	P value
CBCL, internalizing score	51.3±3.1	61.4±7.5	−5.34	33	<0.001
CBCL, externalizing score	51.0±3.3	66.8±10.9	−5.99	33	<0.001
Continuous performance test					
Omission error	44.5±5.6	91.2±40.0	−5.18	36	<0.001
Commission error	47.9±9.9	103.2±43.6	−5.52	36	<0.001
Response time, mean	55.4±11.1	53.1±19.9	0.44	36	0.659
Response time, SD	56.4±12.4	109.7±46.1	−4.99	36	<0.001
Digit span, forward	11.5±2.7	10.3±3.1	1.28	37	0.209
backward	6.8±2.0	4.7±2.1	3.14	37	0.003
Total	18.2±4.4	14.9±4.1	2.39	37	0.022
Finger window, forward	17.0±3.5	12.8±5.8	2.74	38	0.009
backward	15.9±4.7	9.1±5.8	4.04	38	<0.001
Total	32.8±6.9	21.9±10.8	3.80	38	0.001
COWAT, category	26.0±5.7	23.6±7.7	1.10	38	0.279
letter	25.6±10.7	21.6±10.6	1.20	38	0.237

T-test.

The higher the subscale score of CBCL, the more problematic. *The better cognitive performance means lower continuous performance test scores, and higher digit span and finger window test scores.

### Functional Connections Showing Age-by-group Interaction among All Possible Functional Connections

In linear regression analyses, two among 66 functional connections showed significant age-by-group interaction after correction for multiple comparisons: Sensory/motor – SN, and pDMN/prec – aDMN connections.

In the functional connectivity between Sensory/motor and SN, there was no significant difference between the proposed and the selected models (F = 0.80, p = 0.56). The proposed model was, however, better fitted than non-interaction one (F = 11.89, p = 0.002). In this proposed model, there was a significant age-by-group interaction in Sensory/motor-SN (Table 3):
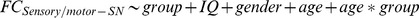



**Table 3:** Summary of the result of regression analysis of functional connectivity in RSFN pairs showing significant group-by-age interaction.

	Estimate	SE	t	P value	q value	CI		
						low		upper
aDMN-pDMN/prec								
(Intercept)	−2.91	0.59	−4.95	<0.001	0.001	−4.00		−1.82
Group	1.71	0.39	5.24	<0.001	<0.001	1.14		2.37
IQ	0.02	0.003	4.39	<0.001	0.008	0.01		0.02
gender	2.98	1.17	2.55	0.016	0.43	0.84		5.37
Age	0.12	0.03	4.62	<0.001	0.005	0.07		0.18
IQ×gender	−0.02	0.006	−2.47	0.020	0.99	−0.03		−0.004
Group×age	−0.15	0.03	−5.55	<0.001	<0.001	−0.21		−0.11
Gender×age	−0.11	0.06	−1.99	0.056	0.38	−0.22		−0.01
Sensory/motor-SN								
(Intercept)	0.005	0.54	0.01	0.99	0.80		−1.09	1.00
Group	0.75	0.32	2.39	0.02	0.20		0.28	1.44
IQ	0.05	0.03	1.66	0.11	0.59		−0.01	0.004
gender	−0.002	0.003	−0.55	0.59	0.58		−0.23	0.25
age	0.03	0.13	0.22	0.83	0.72		−0.003	0.10
Group×age	−0.09	0.03	−3.45	0.002	0.03		−0.15	−0.05

SE: standard error, CI: confidence interval estimated with bootstrapping, q value: calculated for the correction for multiple comparisons using false discovery rate; aDMN-pDMN/prec: Regression: F = 6.72, df = 7, p<0.001; Residual: df = 27, SE = 0.17; Multiple R-squared: 0.62, Adjusted R-squared: 0.53.

Sensory/motor-SN: Regression: F = 4.50, df = 5, p = 0.003; Residual: df = 31, SE = 0.20; Multiple R-squared: 0.42, Adjusted R-squared: 0.33.

**Figure 2:**
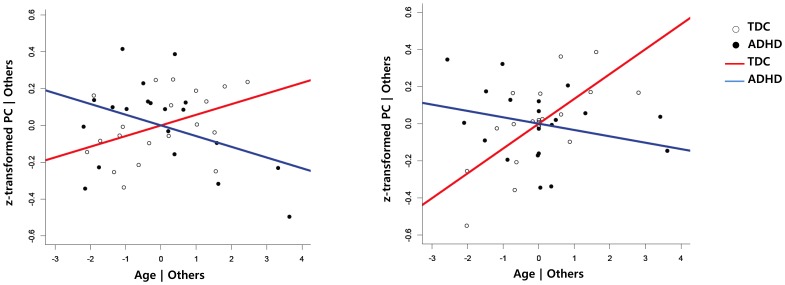
Added-variable (partial regression) plots between age and the functional connectivities. To present the age-by-group interaction, the plots from each group were overlayed. Left: Relationship between age and SN-sensory/motor networks after having adjusted the effect of group, IQ, gender and age. Right: Relationship between age and pDMN/prec – aDMN connectivity after having adjusted the effect of group, IQ, gender, age, IQ by gender and gender by age (right). PC = Partial correlation coefficient, Blue line = ADHD, Red line = TDC.

There was no significant effect on the multiple linear regression analysis of each group. In the functional connectivity between the Sensory/motor network and SN, the slope gradually increased by getting older in TDC group, but decreased in the ADHD group (Figure 2-A). This functional connectivity may be the same at birth (age = 0) and its difference was gradually increased by aging.

In the functional connectivity between pDMN/prec and aDMN, the selected model using the bidirectional elimination approach better fitted the data than the proposed model (F = 3.69, p = 0.04) and also better than the non-interaction model (F = 11.30, p<.0001). The selected model was:
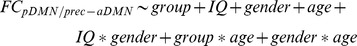



In this selected model, there were significant main effects on intercept, group, and IQ, as well as age, by group interaction in the aDMN-pDMN/prec connection after correction for multiple comparisons (Table 3). In the multiple linear regression analysis for each group, the TDC group showed significant effects from intercept (Estimates = −3.19, t = −3.64, p = 0.003), IQ (Estimates = 0.02, t = 3.17, p = 0.007) and age (Estimates = 0.13, t = 3.66, p = 0.003). However, the ADHD group showed significant effects from IQ (Estimates = 0.01, t = 2.56, p = 0.03) and gender (Estimates = 4.89, t = 2.27, p = 0.04), but not age (Estimates = −0.03, t = −1.28, p = 0.23), in the functional connectivity between pDMN/prec and aDMN. In the functional connectivity between pDMN/prec and aDMN, the slope increased with age in the TDC group, but not in the ADHD group (Figure 2B). However, the significant effects disappeared after correction for multiple comparisons with FDR (q <0.05). According to the resulting regression formula, this functional connectivity may be higher in ADHD subjects than in TDC subjects at age 0, but it may be the same in both groups having IQ 100 at ages 13.78 in males and 13.92 in females. In the non-interaction model, there was no main effect of group after correction for multiple comparisons. Furthermore, there was no functional connection showing better fit of non-interaction than with the proposed models.

### rsRAIs Showing Age-by-group Interaction and Its Relationship with aDMN-pDMN/prec Connection

**Figure 3:**
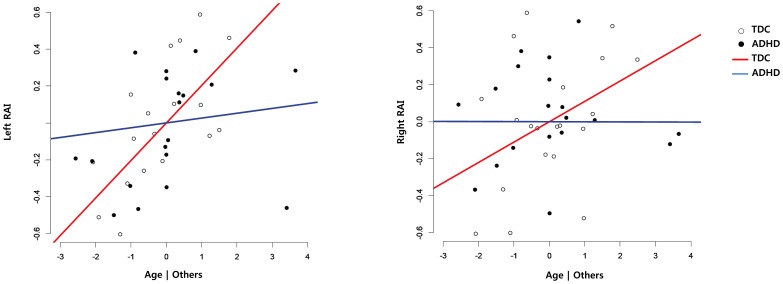
Added-variable (partial regression) plots between age and the resource allocation index in resting state (rsRAI). Relationship between age and rsRAI in left- or right-side CEN-SN-pDMN/prec connections after having adjusted the effect of group, IQ, gender and age. To present the age by group interaction, the plots acquired from each group were overlayed. Blue line = ADHD, Red line = TDC, CEN = central executive network, SN = salience network, pDMN/prec = posterior DMN.

Medication-naïve ADHD subjects failed to show the age-related increment of rsRAIs in bilateral CEN-SN-pDMN/prec connections (age by group interaction, right: t = −3.41, p = 0.002; left: t = −2.82, p = 0.008), which was shown in the TDC group. The bilateral rsRAIs survived after Bonferroni correction at the level of P<0.0125 (0.05/4). (See Figure 3 and Table 4.) No age-by-group interaction was found in bilateral CEN-SN-aDMN (right: t = 0.05, p = 0.689; left t = 0.05, p = 0.839). These results suggest resource allocation by SN to pDMN/prec; and that CEN has a developmental property which was not found in the medication-naïve ADHD group.

**Table 4:** Summary of the result of regression analysis of resource allocation index in resting state (rsRAIs) showing a significant group-by-age interaction.

	Estimate	SE	t	P value	CI	
					low	upper
rFP-SN-pDMN/prec						
(Intercept)	−2.31	0.97	−2.38	0.023	−4.23	−0.56
Group	1.93	0.57	3.39	0.002*	0.91	3.03
IQ	0.01	0.006	2.01	0.052	0	0.02
gender	−0.48	0.25	−1.93	0.061	−1.11	−0.16
age	0.15	0.05	3.00	0.005*	0.08	0.27
Group×age	−0.17	0.05	−3.41	0.002*	−0.26	−0.08
lFP-SN-pDMN/prec						
(Intercept)	−3.63	0.94	−3.85	<0.001*	−5.66	−2.09
Group	1.55	0.55	2.81	0.008*	0.32	2.35
IQ	0.02	0.005	3.50	0.001*	0.01	0.029
gender	−0.55	0.24	−2.28	0.029	−1.38	0.44
age	0.19	0.05	3.72	<0.001*	0.13	0.32
Group×age	−0.14	0.05	−2.82	0.008*	−0.21	−0.03

SE: standard error, CI: confidence interval estimated with bootstrapping (type = BCa: bias-corrected, accelerated confidence intervals [54]);

Bonferroni corrected P<0.0125; rFP-SN-pDMN/prec: Regression: F = 3.14, df = 5, p = 0.02; Residual: df = 34, SE = 0.38; Multiple R-squared: 0.31, Adjusted R-squared: 0.21; lFP-SN-pDMN/prec: Regression: F = 4.76, df = 5, p = 0.002; Residual: df = 34, SE = 0.37; Multiple R-squared: 0.41, Adjusted R-squared: 0.33.

### Correlation of rsRAI with Functional Connection Showing Significant Age by Group Interaction

Partial correlation analysis with age and IQ controlled out at the level of P<0.05, showed that the rsRAI of right CEN-SN-pDMN/prec was positively related with aDMN-pDMN/prec (Rho = 0.413, p = 0.015), not SN-Sensory/motor (Rho = 0.227, p = 0.197), connectivity across all subjects (Table 5). This positive relationship between the rsRAI of right CEN-SN-pDMN/prec and aDMN-pDMN/prec connectivity showed in the TDC group with a trend level (Rho = 0.414, p = 0.099), but was not found in medication-naïve ADHD subjects (Rho = 0.123, p = 0.676). The negative relationship between the rsRAI of left CEN-SN-aDMN and aDMN-pDMN/prec connectivity showed in medication-naïve ADHD subjects with a trend level (Rho = −0.495, p = 0.072).

**Table 5:** Correlation of rsRAI with aDMN-pDMN/prec functional connectivity.

	rCEN-SN-pDMN/prec		lCEN-SN-pDMN/prec		rCEN-SN-aDMN		lCEN-SN-aDMN	
	Rho	P value	Rho	P value	Rho	P value	Rho	P value
All subjects	0.413	0.015	0.227	0.197	−0.023	0.896	−0.301	0.084
TDC	0.414	0.099	0.150	0.567	0.074	0.778	−0.373	0.140
ADHD	0.123	0.676	−0.226	0.438	−0.438	0.117	−0.495	0.072

rsRAI: resource allocation index in resting state; aDMN: anterior default mode network; pDMN/prec: precuneus part of posterior default mode network.

### Correlation of Clinical Variables with Functional Connection or rsRAI Showing Significant Age-by-group Interaction

After controlling for age, IQ and gender effects, the age-related decline of functional connectivity in Sensory/motor – SN was associated with the increased K-ARS total (Rho = −0.43, p = 0.01), inattention (Rho = −0.48, p = 0.005) and hyperactivity (Rho = −0.37, p = 0.03) scoresacross all participants. It was also associated with increased omission (Rho = −0.36, p = 0.04) and commission errors (Rho = −0.50, p = 0.003) in CPT, and with decreased backward (Rho = 0.38, p = 0.03) and total (Rho = 0.37, p = 0.03) T-scores in the Finger window test across all participants. There were no significant relationships in each group.

The increased functional connectivity in pDMN/prec – aDMN was associated with increased verbal (Rho = 0.48, p = 0.003) and total IQ (Rho = 0.48, p = 0.003) across all subjects; when both age and gender were controlled. This relationship was also found in both the ADHD (verbal IQ: Rho = 0.49, p = 0.06; total IQ: Rho = 0.53, p = 0.04) and TDC (verbal IQ: Rho = 0.53, p = 0.02; total IQ: Rho = 0.63, p = 0.006) groups. In the ADHD group, the decreased functional connectivity in pDMN/prec – aDMN was associated with lower body weight (Rho = 0.71, p = 0.005) and with shorter height (Rho = 0.52, p = 0.056) indicating a delay in physical development.

## Discussion

This resting-state fMR study provides an opportunity to identify aberrant developmental trajectories of two RSFN connections: the SN-Sensory/Motor and aDMN-pDMN/prec connections in medication-naïve ADHD children. The SN-Sensory/Motor connection was associated with behavioral or cognitive symptoms. The aDMN-pDMN/prec connection was associated with body weight, height and IQ. Additionally, these abnormalities were related with deficits in the development of resource allocation by the SN in medication-naïve ADHD children. To the best of our knowledge, this is the first report of the aberrant development of resource allocation by SN, and of its relationship with aberrant development of the inter-DMN connectivity in medication-naïve ADHD children.

Although the cellular basis of the altered developmental trajectory of the functional connection between specific RSFNs has yet to be established, the delayed or failed development of the between-RSFN connections in medication-naïve ADHD children may be attributed to the slower maturation of the brain network system including neuronal synaptic pruning [40], myelination [41] or microglial interaction with synapses [42]. These two RSFN connections might be the neuroimaging biomarker for the brain development of, and the treatment for, ADHD children.

### Developmental Abnormalities in the Interaction between SN and Sensory/motor Networks

In a narrow sense, the SN includes dACC, AI and PPC. However, it is also named the cingulo-opercular network. The cingulo-opercular network is thus a variant name of the SN, which anchored by the AI and ACC. The ventral attentional network includes the temporo-parietal junction, the supra-marginal gyrus, frontal operculum and AI [43]. Studies on seed-based functional connectivity [44] and causality [45] indicated that the ventral attentional network was closely associated with the cingulo-opercular network. The ventral attentional network signals task-transition when environmental stimuli call for a change in an ongoing task [43]. In ICA with low dimension for resting-state fMRI studies like this one, both salience and ventral attentional networks appear as a single one anchored more by the frontal opercular region (including insula and parietal region), than the ACC [25]. Therefore, in the present study, we called the overlapped network the SN.

Menon et al. (2010) proposed a model of dynamic bottom-up and top-down interaction underlying attentional control. According to their model, first, deviant stimulus detected by primary sensory areas is transmitted to AI and ACC. Second, AI and ACC generate a ‘top-down’ control signal which is transmitted to the primary sensory area and other neocortical regions including the dorsolateral prefrontal cortex, temporo-parietal area and premotor cortex. Third, the neocortical regions respond to the attentional shift. Finally, the ACC facilitates response selection and motor response. Thus, functional connection between SN and sensory/motor networks reflects both attentional and motor responses to significant stimuli.

In the present study, the functional connection between SN and sensory/motor network declined in medication-naïve ADHD subjects across age, while it increased in the TDC subjects. Furthermore, the decreased functional connection was associated with increased behavioral symptom scores, especially with inattentive symptoms, and with poorer visual cognitive abilities such as the finger-window-backward task representing visual working memory ability and omission/commission errors of visual CPT across all subjects. In a study using regions of interest in key nodes of the SN, CEN, and DMN; the functional connectivity of the right fronto-insular cortex with the ACC, right dorsolateral prefrontal cortex, and posterior cingulate cortex; were greater in adults than children [46]. In a recent 33-year follow-up study in adults with childhood ADHD, decreased fractional anisotropy in regions involved in sensorimotor, as well as high-level cognitive functions including, in particular, visual processing were reported [47]. Thus, our findings showing developmental delay of the sensory/motor-SN functional connectivity is in line with results from previous studies using functional [46] or anatomical [47] connectivity. Interestingly, in our multiple linear regression analysis for SN-sensory/motor network connection, there was no difference in intercept (Table 3). This suggests that the abnormal development of the SN-sensory/motor network in medication-naïve ADHD may be not inborn but acquired.

### Developmental Abnormalities in the Interaction between Anterior And Posterior DMNs

Medication-naïve ADHD subjects also failed to show age-related increment in the functional connection between pDMN/prec and anterior DMN. The decreased functional connection between posterior and anterior DMNs was associated with decreased IQ across all subjects, as well as in each group. The anterior and posterior DMNs integrate into a cohesive, interconnected network during development [48]. This maturation of the default network appeared to be delayed or disrupted in ADHD children (7–16 years) scanned after a minimum washout of five half-live of psychostimulant [10]. In addition, the decreased aDMN-pDMN connectivity was also reported in adults with ADHD [11]. The cingulum bundle, a possible anatomical connection between the anterior and posterior DMN, continues to develop into adulthood [49], [50]. Aberrant anatomical connectivity in the cingulum bundle has been reported in patients with ADHD [e.g., increased ADC [51] and reduced FA [52], [53]]. The previous studies compared group mean bivariate functional connectivity among *a priori* ROIs which was measured with simple Pearson correlation analysis in patients having medication [10], [11]. Here, in the present study, we confirmed the delayed maturation of the default network connection in medication-naïve ADHD children using data-driven ROIs and partial correlation analysis.

In the regression analyses, to investigate age-related changes of functional connectivity between pDMN/prec and anterior DMN, there were significant main effects of intercept, group, age, and IQ; as well as group-by-age interaction (Table 3). In the multiple linear regression analysis for each group, the main effect of age was significant in the TDC, but not in the medication-naïve ADHD, group. Our findings of both higher functional connectivity at birth (significant main effect of intercept) and the failure of normal development of the aDMN-pDMN/prec connection (Table 3) indicate that segregation within each network, as well as integration between two networks, may be disrupted or delayed in medication-naïve ADHD children, in contrast to TDC subjects. Our results suggest that age-related change should be considered in the study of developing subjects. In further studies with large sample size, it should be investigated whether the aberrant development of this connection is disrupted or delayed in medication-naïve ADHD subjects over a wide range of age, and whether it can be modulated with the treatment.

### Developmental Abnormalities in Resource Allocation and its Relationship with aDMN-pDMN/prec Connectivity

As we predicted, medication-naïve ADHD children failed to show age-related increment of rsRAI in bilateral CEN-SN-pDMN/prec; which was found in the TDC group (Table 4). In a previous study, a significantly negative correlation of age with resting-state functional connectivity between the dACC (part of the SN), and the posterior cingulate cortex (a principle member of pDMN/prec) was reported in TDC subjects. Furthermore, this negative correlation was not found in boys with ADHD [12]. This previous result suggestedin aberrant development of the SN-pDMN/prec in ADHD subjects. Results from cortical thickness studies also suggested aberrant brain development, including in the resource allocation-related regions [4], [5]. In the 33 year follow up study in adult ADHD established in childhood; compared with normal controls, patients had thinner cortex in the bilateral parietal lobes, right precuneus, and precentral gyri; which suggest aberrant development [4]. In a recent longitudinal study, it was reported that bilateral cingulate and medial prefrontal cortices (a part of the SN); and the right dorsolateral prefrontal cortex (a part of the CEN), were thinner in the group in which ADHD persisted into adulthood [5]. These results from previous studies showing the aberrant development in resource allocation related regions are consistent with our rsRAI findings in the medication-naïve ADHD group. Thus, the neuroimaging correspondence of attentional lapses [17] may not be confined to the aberrant interaction between the task-negative DMN, and the task-positive attentional network [18], but should be extended to the rsRAI related networks (CEN-SN-pDMN). Also, our result showing the positive relationship between the rsRAI of right CEN-SN-pDMN/prec and aDMN-pDMN/prec connectivity, suggests that the tighter aDMN-pDMN/prec connection is, the more mature the resource allocation system of right CEN-SN-pDMN/prec in normal development. In contrast, our results showing the negative relationship between the rsRAI of left CEN-SN-aDMN and aDMN-pDMN/prec connectivity suggests the aberrant interaction between resource allocation and DMN systems in medication-naïve ADHD children although the functional meaning of left side rsRAI including aDMN remains to be clarified.

The response to treatment can modulate the developmental trajectory of an ADHD brain. The remitted ADHD, in contrast to persistent ADHD, showed a less-steep slope of age-related thinning of the cerebral cortex, especially in the medial prefrontal/cingulate and dorsolateral prefrontal cortex [5]. If the aberrant functional connection can be restored by treatment, early intervention as soon as possible may be beneficial in terms of brain development in those with ADHD. However, further studies are needed to answer the question whether our results suggesting delayed maturation of the two functional connections, is a treatment target or a response predictor in medication-naïve ADHD subjects.

IQ is one of main aspects of brain function which can affect aDMN-pDMN/prec connection. When the multiple linear regression analysis was performed for each group, the main effect on IQ was significant in both groups. We also found a positive relationship between IQ and this connectivity across all subjects, as well as in each group. Thus, our results suggest that those with higher IQ have stronger aDMN-pDMN/prec connection in both groups. In further study, an IQ-matched sample may be needed.

In addition, we should comment on several limitations in the present study. First, age-related development of functional connectivity could have a nonlinear trajectory. Unfortunately, the nonlinear trajectory issue was not able to be investigated because of research funding constraints for large sample size for neuroimaging studies. Second, our results could be affected by heterogeneity in terms of gender or ADHD subtypes. Third, the number of ROIs delineated by ICA can vary according to user decisions about dimensionality. In this study, we used the best-fit result provided by MELODIC in FSL. Although our approach with relatively low dimensionality (12 by 12 interactions) focused on the demonstration of brain status as the interaction among large scale RSFNs, ICA analysis with high dimensionality may be helpful to investigate within-network information. These limitations should be considered in further large-sample studies of medication-naïve ADHD subjects. The false discovery rate used in the present study may not be the best option to correct for multiple comparisons.

In summary, this resting state fMRI study to investigate developmental differences in functional connectivity among RSFNs, has shown that medication-naïve ADHD may have delayed the maturation of the two functional connections, SN-Sensory/Motor and aDMN-pDMN/prec. Interventions that enhance the functional connectivity of these two connections may get attention as potential therapeutic or preventive options both for ADHD, and for typically-developing children exposed to a highly competitive environment.

## Supporting Information

Figure S1 **Artifactual components.**
(DOCX)

Figure S2 **RSFNs from separate ICA for each group.** Spatial correlation coefficients between corresponding RSFNs from each group from RSFN1 to RSFN12∶0.67, 0.81, 0.47, 0.77. 0.70, 0.73, 0.72, 0.73, 0.81, 0.68, 0.79, 0.65.
(DOCX)

Table S1 **Resting state related independent components (RSFNs).** N: network; S: system; DMN: default mode network; SMA: supplementary motor area; Inf: inferior; Sup: superior; C: cortex; G: gyrus; L: left; R: right; B: bilateral.
(DOCX)

Table S2 **Functional connectivity among RSFNs: mean correlation coefficients.** ^a^Statistically significant age by group interaction at q <0.05. Right upper side: Healthy TDC group; Left lower side: ADHD group. From left-upper to right-lower part: RSFN1: Frontal; RSFN2: Sensory/motor; RSFN3: Salience (SN, Ventral attentional); RSFN4: Right central executive (rCEN); RSFN5: Left central executive network (lCEN); RSFN6: Dorsal attentional (dAtt); RSFN7: V1; RSFN8: V1/V2; RSFN9: Extrastriate; RSFN10: a temporooccipital part of posterior DMN (pDMN/TO); RSFN11: a precuneus part of Posterior default mode (pDMN/prec); RSFN12: Anterior default mode (aDMN). Age by group interaction after the correction for multiple comparison using FDR: RSFN 2– RSFN 3 pair (p = 0.002, q = 0.0116); RSFN 11– RSFN 12 pair (p = 0.0016, q = 0.0415).
(DOCX)

Table S3 **The number of subjects across age ranges.** Two-side fisher’s exact test.
(DOCX)
