# Supplementary material for: Aberrant Development of Functional Connectivity among Resting State-Related Functional Networks in Medication-Naïve ADHD Children
Source: PLoS One. 2013 Dec 26;8(12):e83516. doi: 10.1371/journal.pone.0083516 (PMC3873390; doi:10.1371/journal.pone.0083516)
Supplement: Figure S1 — Artifactual components. (DOCX) [file pone.0083516.s001.docx]

**Figure S1. Artifactual components.**
