# Supplementary material for: Aberrant Development of Functional Connectivity among Resting State-Related Functional Networks in Medication-Naïve ADHD Children
Source: PLoS One. 2013 Dec 26;8(12):e83516. doi: 10.1371/journal.pone.0083516 (PMC3873390; doi:10.1371/journal.pone.0083516)
Supplement: Figure S2 — RSFNs from separate ICA for each group. Spatial correlation coefficients between corresponding RSFNs from each group from RSFN1 to RSFN12∶0.67, 0.81, 0.47, 0.77. 0.70, 0.73, 0.72, 0.73, 0.81, 0.68, 0.79, 0.65. (DOCX) [file pone.0083516.s002.docx]

**Figure S2. RSFNs from separate ICA for each group.**

Spatial correlation coefficients between corresponding RSFNs from each group from RSFN1 to RSFN12: 0.67, 0.81, 0.47, 0.77. 0.70, 0.73, 0.72, 0.73, 0.81, 0.68, 0.79, 0.65.
