# Supplementary material for: Aberrant Development of Functional Connectivity among Resting State-Related Functional Networks in Medication-Naïve ADHD Children
Source: PLoS One. 2013 Dec 26;8(12):e83516. doi: 10.1371/journal.pone.0083516 (PMC3873390; doi:10.1371/journal.pone.0083516)
Supplement: Table S1 — Resting state related independent components (RSFNs). N: network; S: system; DMN: default mode network; SMA: supplementary motor area; Inf: inferior; Sup: superior; C: cortex; G: gyrus; L: left; R: right; B: bilateral. (DOCX) [file pone.0083516.s003.docx]

**Table S1. Resting state related independent components (RSFNs).**

|  |  |  |  | MNI coordinates | | | Z values | P values |
| --- | --- | --- | --- | --- | --- | --- | --- | --- |
| RSFN | Regions | voxels | max(Z) | x | y | z |  |  |
| 1 | R. Mid. Frontal G. | 3556 | 10.60 | 30 | 46 | 28 |  |  |
|  | L. Mid. Frontal G. |  | 9.74 | -26 | 26 | 48 |  |  |
|  | R. Sup. Frontal G. |  | 9.16 | 26 | 10 | 60 |  |  |
|  | L. Sup. Frontal G. |  | 9.16 | -26 | 38 | 40 |  |  |
|  | L. Cerebellum(Crus 1) | 83 | 3.61 | -34 | -74 | -28 |  |  |
|  | L. Cerebellum(Crus 2) |  | 2.58 | -30 | -70 | -44 |  |  |
| 2 | R. Angular G. | 3234 | 12.90 | 42 | -62 | 36 |  |  |
|  | R. Inf. Temporal G. | 161 | 3.31 | 54 | -18 | -24 |  |  |
|  | L. Angular G. | 133 | 4.47 | -42 | -66 | 36 |  |  |
|  | L. Postcentral G. | 132 | 4.36 | -54 | -18 | 32 |  |  |
|  | L. Cerebellum(VII) | 95 | 3.60 | -30 | -66 | -44 |  |  |
| 3 | R. Mid. Cingulate C. | 1712 | 19.20 | 2 | -34 | 52 |  |  |
|  | L. Paracentral Lobule |  | 13.50 | -14 | -38 | 64 |  |  |
|  | R. Precentral G. |  | 12.80 | 18 | -30 | 68 |  |  |
|  | R. Postcentral G. |  | 10.80 | 22 | -42 | 64 |  |  |
| 4 | R. Precuneus | 3353 | 15.10 | 10 | -74 | 44 |  |  |
|  | L. Sup. Parietal lobule |  | 14.90 | -10 | -78 | 44 |  |  |
|  | R. Mid. Occipital G. |  | 11.90 | 38 | -78 | 24 |  |  |
|  | R. Cerebellum(X) | 94 | 4.13 | 18 | -38 | -44 |  |  |
|  | R. ParaHippocampal G. |  | 2.44 | 26 | -26 | -24 |  |  |
|  | R. Inf. Frontal G.(p. Triangularis) | 80 | 2.88 | 34 | 26 | 24 |  |  |
| 5 | L. Rolandic Operculum | 1857 | 12.30 | -42 | -6 | 4 |  |  |
|  | L. SupraMarginal G. |  | 11.00 | -54 | -26 | 16 |  |  |
|  | R. Insula Lobe | 1473 | 12.50 | 46 | -2 | 4 |  |  |
|  | R. SupraMarginal G. |  | 10.30 | 58 | -26 | 20 |  |  |
|  | R. Cuneus | 292 | 5.51 | 14 | -70 | 28 |  |  |
|  | L. Cuneus |  | 4.27 | -10 | -78 | 20 |  |  |
| 6 | L. Precuneus | 1458 | 17.70 | 2 | -62 | 28 |  |  |
|  | L. Post. Cingulate C. |  | 14.60 | -2 | -42 | 24 |  |  |
| 7 | L. Cerebellum(VI) | 2310 | 12.00 | -34 | -66 | -20 |  |  |
|  | L. Sup. Occipital G. |  | 9.78 | -18 | -90 | -4 |  |  |
|  | R. Lingual G. |  | 9.71 | 18 | -74 | -12 |  |  |
|  | R. Fusiform G. |  | 9.14 | 38 | -66 | -16 |  |  |
| 8 | L. Inf. Parietal Lobule | 1432 | 11.00 | -38 | -42 | 44 |  |  |
|  | R. Inf. Parietal Lobule | 1287 | 12.30 | 50 | -34 | 48 |  |  |
|  | R. Sup. Parietal Lobule |  | 6.02 | 22 | -70 | 48 |  |  |
|  | R. Cerebellum(VIII) | 83 | 3.05 | 14 | -66 | -48 |  |  |
|  | R. Cerebellum(Crus 2) |  | 2.99 | 38 | -70 | -48 |  |  |
| 9 | L. Mid. Occipital Gyrus | 2985 | 12.10 | -34 | -66 | 32 |  |  |
|  | L. Precentral G. |  | 7.18 | -50 | 6 | 32 |  |  |
|  | L. Inf. Frontal G.(p. Triangularis) |  | 6.91 | -46 | 14 | 32 |  |  |
|  | L. Mid. Cingulate C. | 87 | 4.45 | -2 | -42 | 36 |  |  |
|  | L. Caudate Nucleus | 76 | 2.86 | -18 | -6 | -16 |  |  |
|  | L. Putamen |  | 2.51 | -30 | -18 | 4 |  |  |
| 10 | R. Mid. Temporal G. | 1772 | 12.20 | 54 | -54 | 12 |  |  |
|  | R. Sup. Temporal G. |  | 9.96 | 58 | -18 | 0 |  |  |
|  | L. Mid. Temporal G. | 1141 | 10.00 | -50 | -54 | 12 |  |  |
|  | L. Sup. Temporal G. |  | 5.06 | -54 | -2 | -4 |  |  |
|  | R. Cerebellum(IV-V) | 123 | 4.78 | 18 | -42 | -20 |  |  |
|  | R. Cerebellum(VI) |  | 2.78 | 10 | -66 | -20 |  |  |
| 11 | R. Ant. Cingulate C. | 772 | 12.90 | 6 | 34 | -4 |  |  |
|  | R. Caudate Nucleus |  | 9.87 | 18 | 30 | -4 |  |  |
|  | R. Putamen |  | 8.52 | 22 | 22 | -8 |  |  |
|  | L. Caudate Nucleus |  | 3.65 | -10 | 10 | 0 |  |  |
| 12 | L. Calcarine G. | 1398 | 18.20 | 2 | -74 | 16 |  |  |
|  | R. Calcarine G. |  | 15.50 | 18 | -58 | 8 |  |  |
|  | R. Mid. Occipital G. |  | 2.90 | 42 | -74 | 16 |  |  |
|  | R. Rolandic Operculum | 125 | 3.72 | 50 | -22 | 12 |  |  |

N: network; S: system; DMN: default mode network; SMA: supplementary motor area; Inf: inferior; Sup: superior; C: cortex; G: gyrus; L: left; R: right; B: bilateral
