# Supplementary material for: Aberrant Development of Functional Connectivity among Resting State-Related Functional Networks in Medication-Naïve ADHD Children
Source: PLoS One. 2013 Dec 26;8(12):e83516. doi: 10.1371/journal.pone.0083516 (PMC3873390; doi:10.1371/journal.pone.0083516)
Supplement: Table S2 — Functional connectivity among RSFNs: mean correlation coefficients. aStatistically significant age by group interaction at q <0.05. Right upper side: Healthy TDC group; Left lower side: ADHD group. From left-upper to right-lower part: RSFN1: Frontal; RSFN2: Sensory/motor; RSFN3: Salience (SN, Ventral attentional); RSFN4: Right central executive (rCEN); RSFN5: Left central executive network (lCEN); RSFN6: Dorsal attentional (dAtt); RSFN7: V1; RSFN8: V1/V2; RSFN9: Extrastriate; RSFN10: a temporooccipital part of posterior DMN (pDMN/TO); RSFN11: a precuneus part of Posterior default mode (pDMN/prec); RSFN12: Anterior default mode (aDMN). Age by group interaction after the correction for multiple comparison using FDR: RSFN 2– RSFN 3 pair (p = 0.002, q = 0.0116); RSFN 11– RSFN 12 pair (p = 0.0016, q = 0.0415). (DOCX) [file pone.0083516.s004.docx]

**Table S2 Functional connectivity among RSFNs: mean correlation coefficients.**

|  | RSFN1 | RSFN2 | RSFN3 | RSFN4 | RSFN5 | RSFN6 | RSFN7 | RSFN8 | RSFN9 | RSFN10 | RSFN11 | RSFN12 |
| --- | --- | --- | --- | --- | --- | --- | --- | --- | --- | --- | --- | --- |
| RSFN1 |  | -0.186 | 0.071 | -0.014 | 0.209 | -0.015 | -0.097 | 0.159 | 0.081 | 0.211 | 0.034 | 0.099 |
| RSFN2 | 0.016 |  | 0.311^a^ | -0.062 | -0.074 | 0.231 | -0.115 | 0.082 | 0.143 | 0.152 | 0.095 | -0.112 |
| RSFN3 | -0.026 | 0.173 |  | 0.225 | 0.152 | -0.080 | 0.063 | -0.124 | -0.203 | 0.008 | -0.300 | 0.118 |
| RSFN4 | 0.215 | -0.030 | 0.268 |  | 0.030 | 0.123 | 0.039 | -0.142 | 0.125 | 0.082 | 0.013 | -0.021 |
| RSFN5 | 0.141 | 0.060 | -0.097 | 0.152 |  | 0.153 | 0.100 | -0.221 | -0.001 | -0.011 | 0.065 | -0.067 |
| RSFN6 | -0.091 | 0.221 | -0.088 | 0.246 | 0.237 |  | -0.004 | 0.178 | 0.288 | 0.084 | -0.223 | 0.216 |
| RSFN7 | -0.188 | 0.109 | 0.221 | 0.051 | 0.146 | 0.037 |  | 0.318 | -0.044 | 0.136 | 0.101 | 0.153 |
| RSFN8 | 0.158 | 0.080 | -0.212 | -0.186 | -0.224 | 0.226 | 0.182 |  | 0.146 | -0.118 | 0.094 | -0.090 |
| RSFN9 | 0.022 | 0.193 | -0.284 | -0.055 | 0.025 | 0.090 | -0.011 | 0.242 |  | 0.047 | 0.169 | -0.055 |
| RSFN10 | 0.129 | 0.035 | 0.128 | 0.042 | 0.121 | -0.042 | 0.212 | 0.150 | 0.041 |  | 0.053 | -0.053 |
| RSFN11 | 0.087 | -0.145 | -0.262 | 0.160 | 0.024 | -0.228 | 0.225 | -0.138 | 0.149 | 0.059 |  | 0.092^a^ |
| RSFN12 | 0.167 | 0.062 | 0.152 | -0.050 | 0.014 | 0.020 | 0.048 | -0.097 | -0.062 | 0.088 | -0.044 |  |

**^a^**Statistically significant age by group interaction at q < 0.05.

Right upper side: Healthy TDC group; Left lower side: ADHD group

From left-upper to right-lower part: RSFN1: Frontal; RSFN2: Sensory/motor; RSFN3: Salience (SN, Ventral attentional); RSFN4: Right central executive (rCEN); RSFN5: Left central executive network (lCEN); RSFN6: Dorsal attentional (dAtt); RSFN7: V1; RSFN8: V1/V2; RSFN9: Extrastriate; RSFN10: a temporooccipital part of posterior DMN (pDMN/TO); RSFN11: a precuneus part of Posterior default mode (pDMN/prec); RSFN12: Anterior default mode (aDMN).Age by group interaction after the correction for multiple comparison using FDR: RSFN 2 – RSFN 3 pair (p=0.002, q = 0.0116); RSFN 11 – RSFN 12 pair (p = 0.0016, q = 0.0415)
