# Supplementary material for: Aberrant Development of Functional Connectivity among Resting State-Related Functional Networks in Medication-Naïve ADHD Children
Source: PLoS One. 2013 Dec 26;8(12):e83516. doi: 10.1371/journal.pone.0083516 (PMC3873390; doi:10.1371/journal.pone.0083516)
Supplement: Table S3 — The number of subjects across age ranges. Two-side fisher’s exact test. (DOCX) [file pone.0083516.s005.docx]

**Table S3. The number of subjects across age ranges.**

|  | Age(yr) | | | P value |
| --- | --- | --- | --- | --- |
|  | 6-8 | 9-11 | 12-16 |  |
| Controls | 6 | 10 | 4 | P = 0.72 |
| ADHD | 7 | 7 | 6 |  |

Two-side fisher’s exact test
